# Supplementary material for: Diet in Patients with Myocardial Infarction and Coexisting Type 2 Diabetes Mellitus
Source: Int J Environ Res Public Health. 2023 Apr 7;20(8):5442. doi: 10.3390/ijerph20085442 (PMC10138621; doi:10.3390/ijerph20085442)

**Figures**

Non-dietary lifestyle elements not included in the analysis to maintain the study’s integrity

**Figure 1.** Frequency of alcohol consumption: Total (N=67)


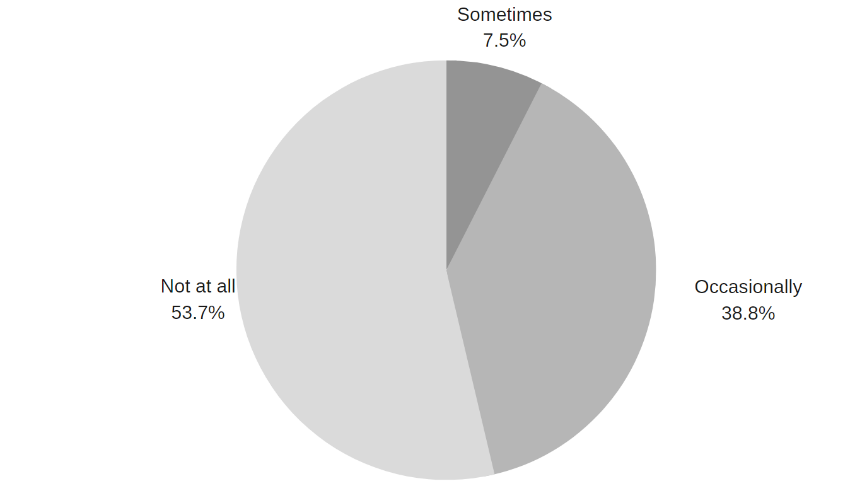


**Figure 2.** Frequency of alcohol consumption: First MI (N=33)


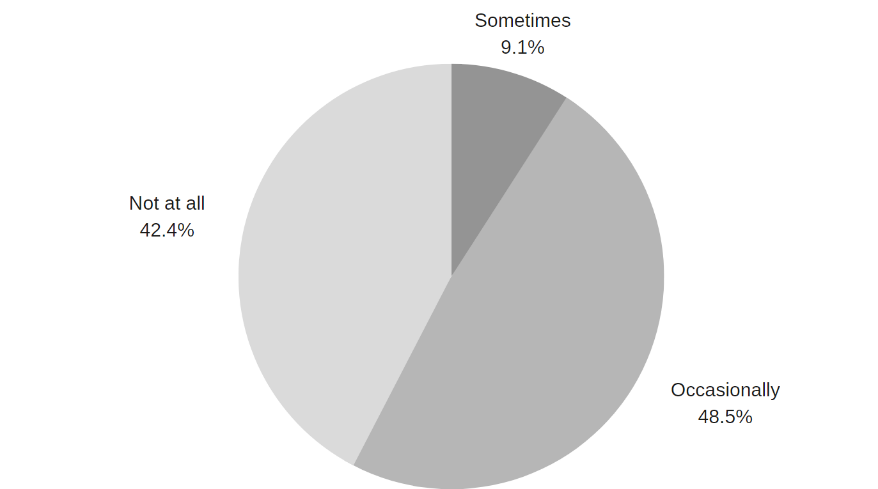


**Figure. 3** Frequency of alcohol consumption: Second MI (N=34)


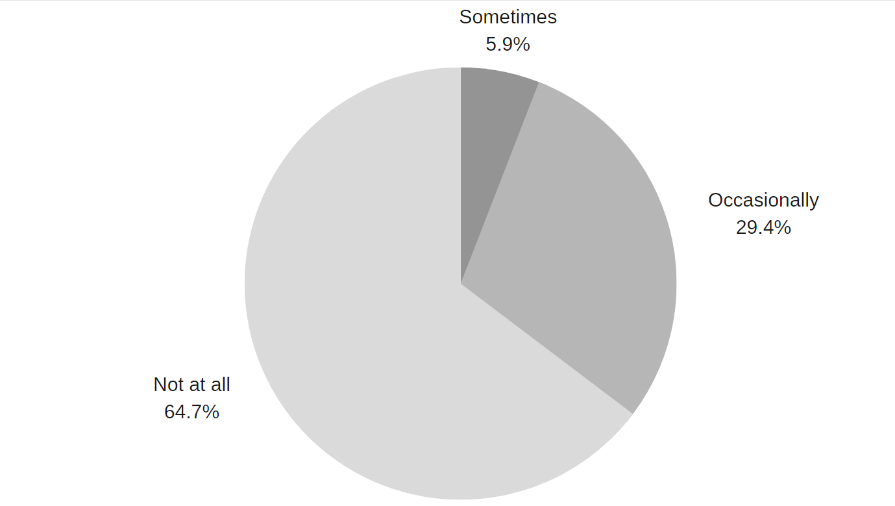


**Figure 4.** Smoking: Total (N=67)


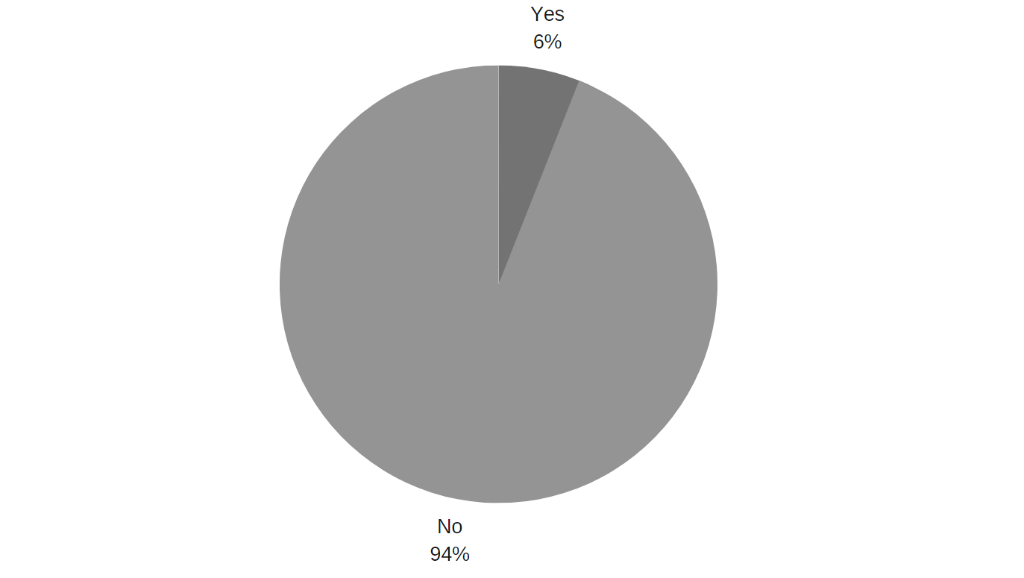


**Figure 5**. Smoking: First MI (N=33)


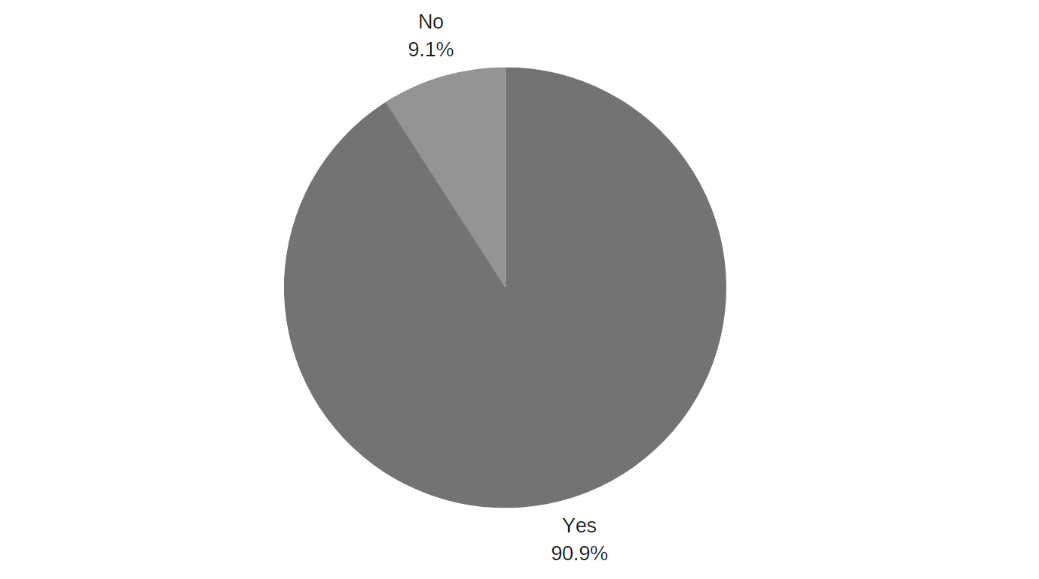


**Figure 6.** Smoking: Second MI (N=34)


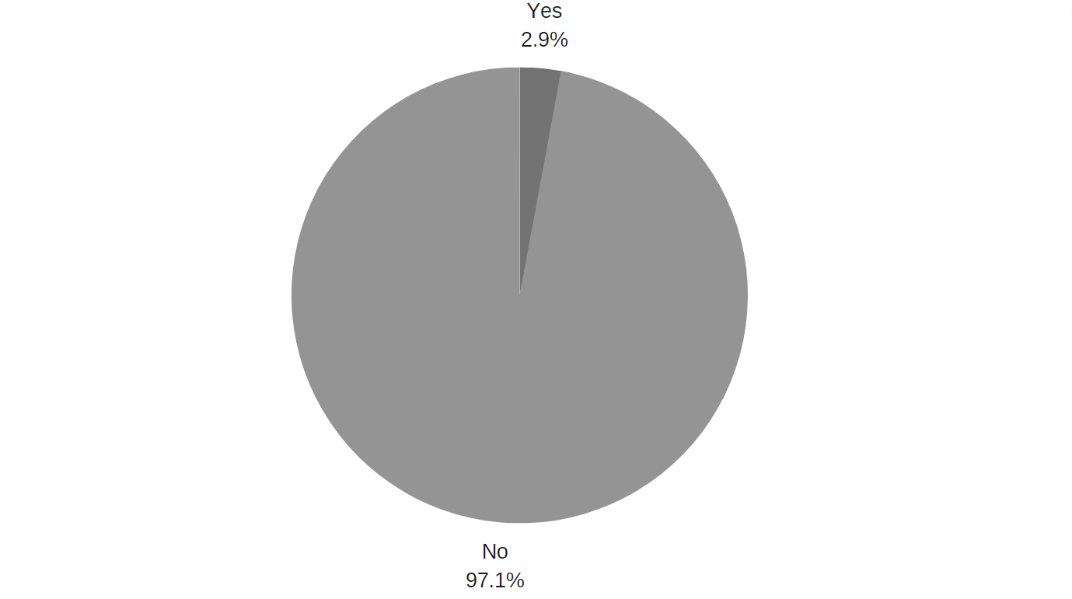


**Figure 7**. Exercise in the past: Total (N=67)


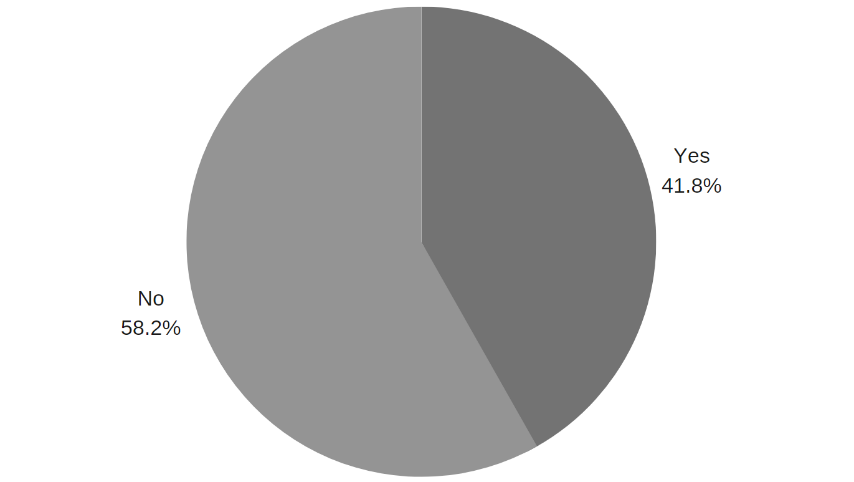


**Figure 8.** Exercise in the past: First MI (N=33)


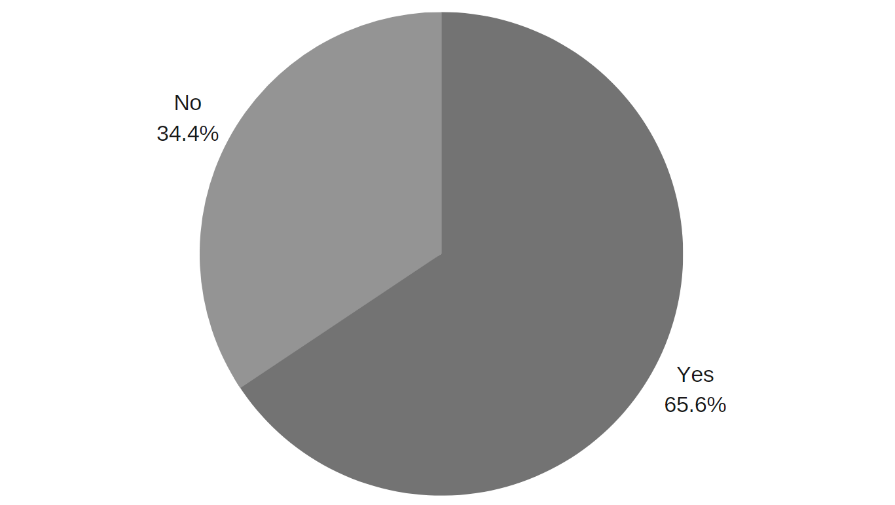


**Figure 9.** Exercise in the past: Second MI (N=34)


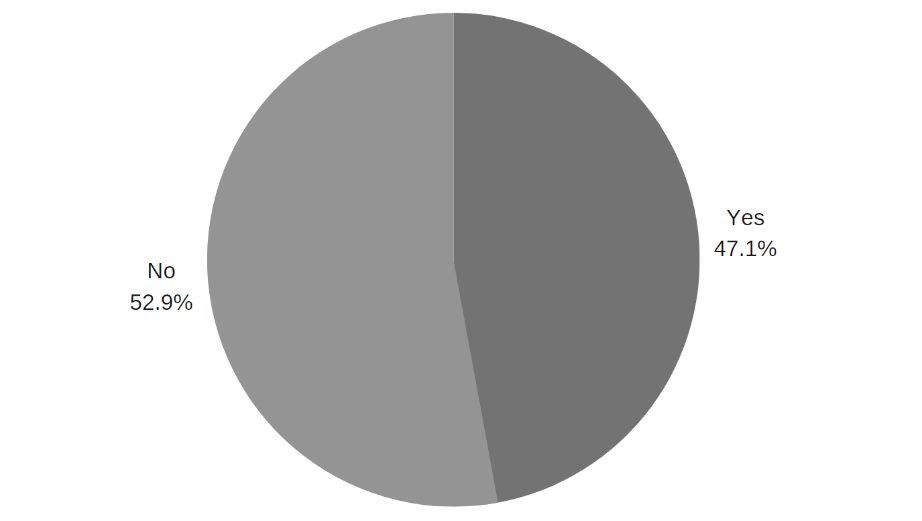


**Figure 10.** Exercise in the future: Total (N=67)


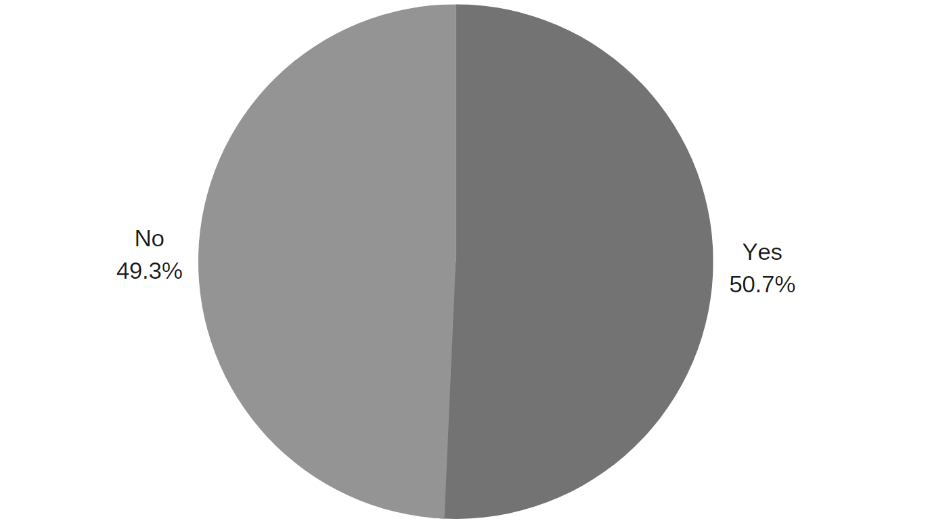


**Figure 11**. Exercise in the future: First MI (N=33)


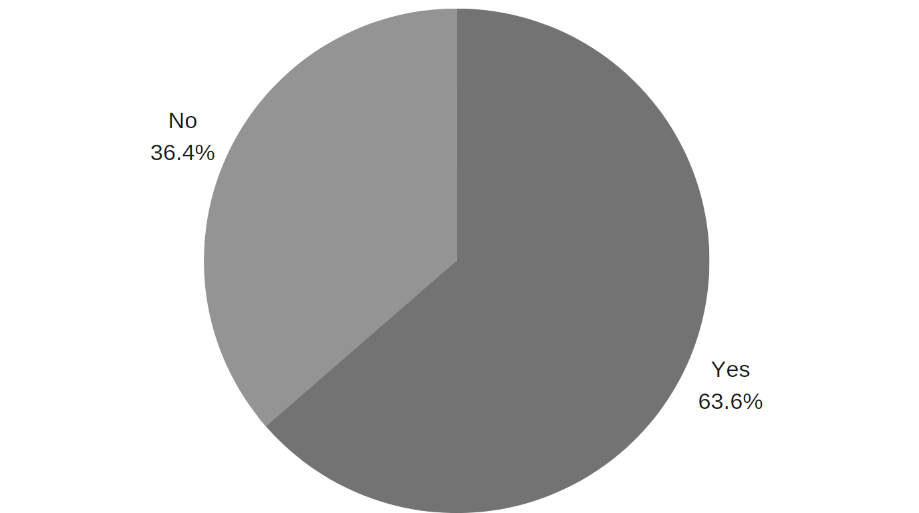


**Figure 12.** Exercise in the future: Second MI (N=34)


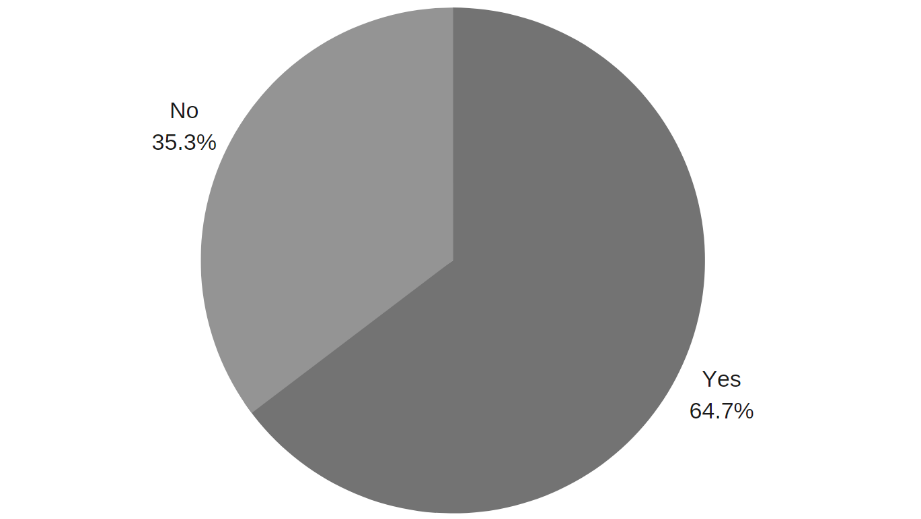

Supplement: Supplementary file 1 [file ijerph-20-05442-s001.zip › ijerph-2220625-supplementary.docx]
