# Supplementary material for: Diet in Patients with Myocardial Infarction and Coexisting Type 2 Diabetes Mellitus
Source: Int J Environ Res Public Health. 2023 Apr 7;20(8):5442. doi: 10.3390/ijerph20085442 (PMC10138621; doi:10.3390/ijerph20085442)

Figures

Non-dietary lifestyle elements not included in the analysis to maintain the study's integrity

Figure 1. Frequency of alcohol consumption: Total (N=67)

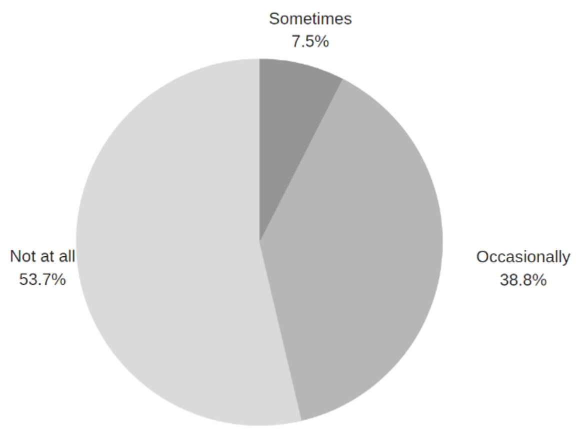

Figure 2. Frequency of alcohol consumption: First MI (N=33)

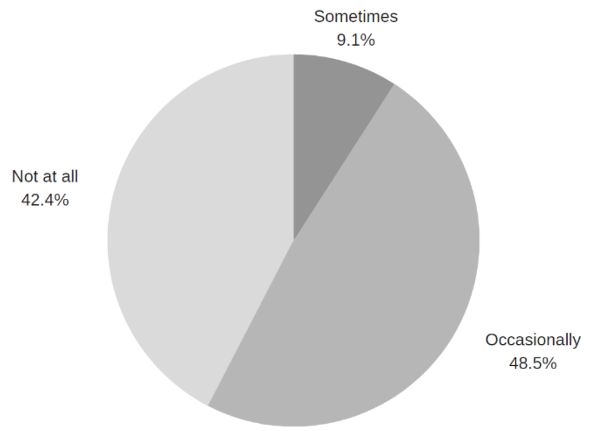

Figure. 3 Frequency of alcohol consumption: Second MI (N=34)

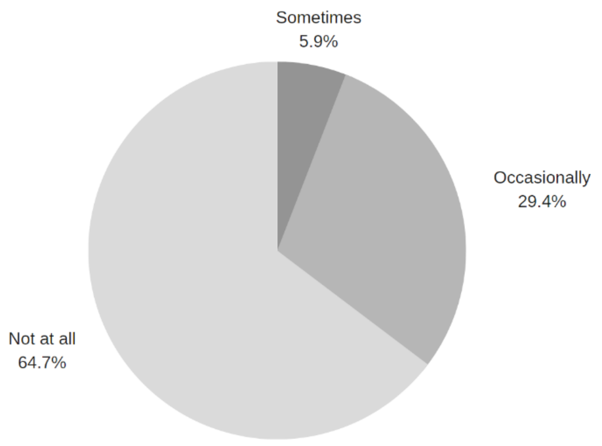

Figure 4. Smoking: Total (N=67)

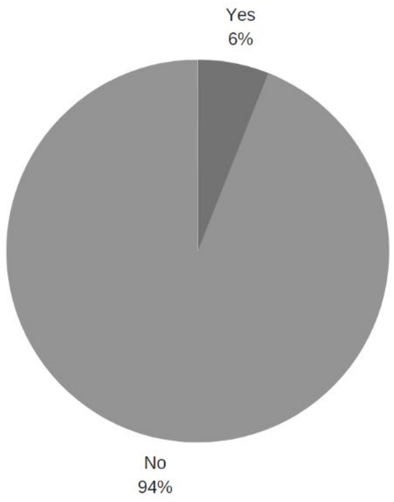

Figure 5. Smoking: First MI (N=33)

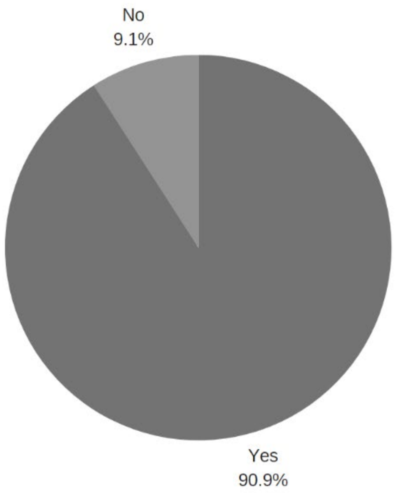

Figure 6. Smoking: Second MI (N=34)

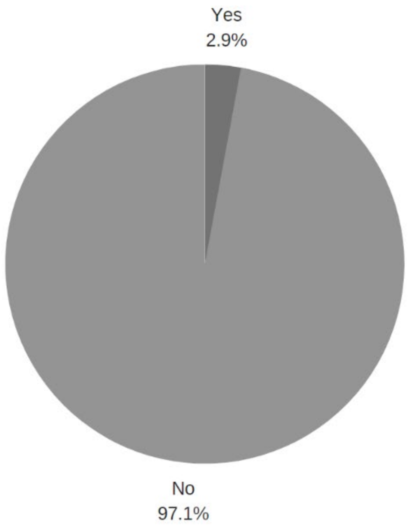

Figure 7. Exercise in the past: Total (N=67)

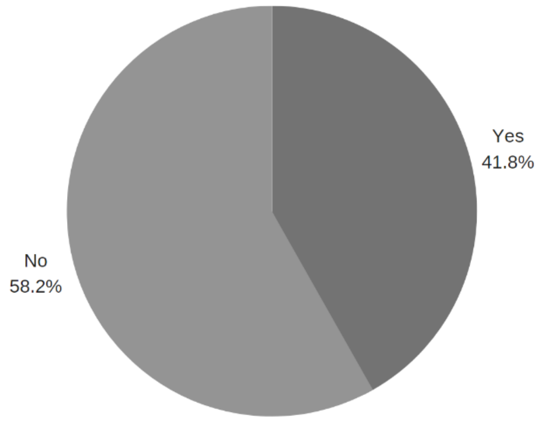

Figure 8. Exercise in the past: First MI (N=33)

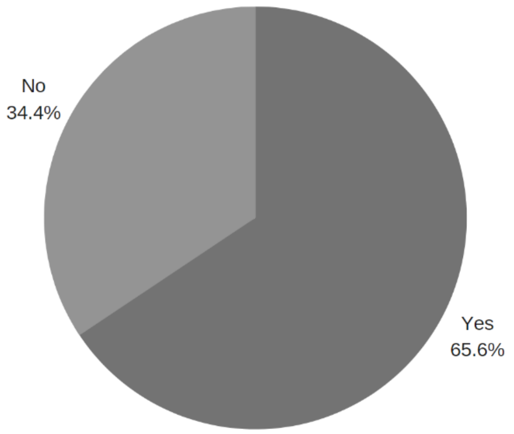

Figure 9. Exercise in the past: Second MI (N=34)

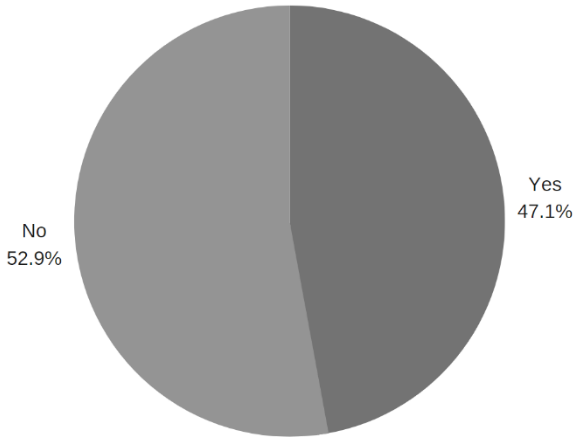

Figure 10. Exercise in the future: Total (N=67)

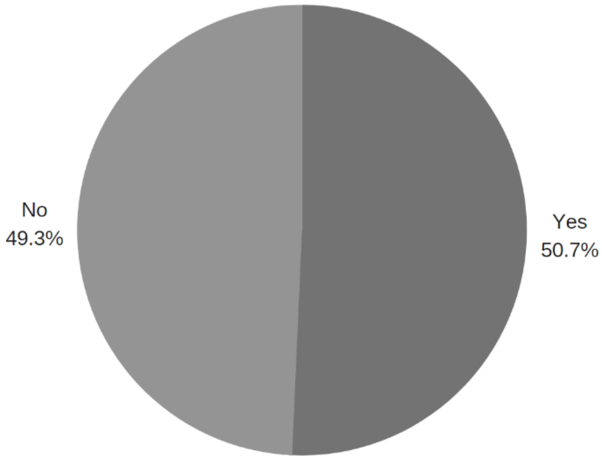

Figure 11. Exercise in the future: First MI (N=33)

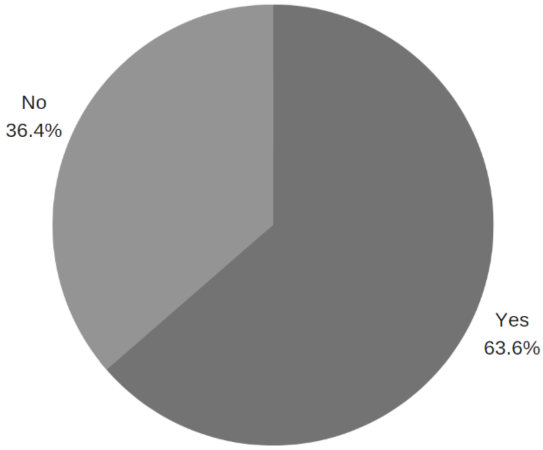

Figure 12. Exercise in the future: Second MI (N=34)

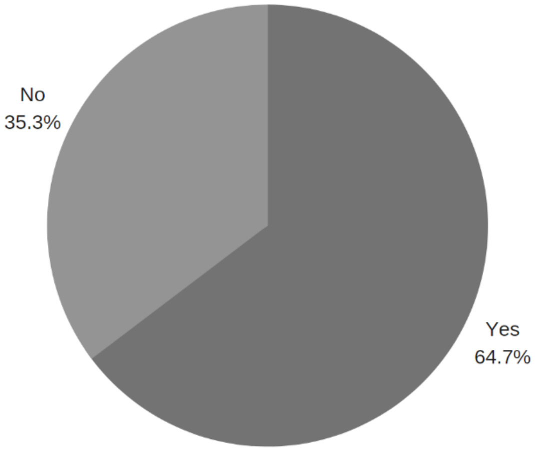

Supplement: Supplementary file 1 [file ijerph-20-05442-s001.zip › ijerph-2220625-supplementary.pdf]
